# Towards Legally Mandated Public Health Benchmarks

**DOI:** 10.34172/ijhpm.2022.7123

**Published:** 2022-12-04

**Authors:** Jochen O. Mierau, Brigit C.A. Toebes

**Affiliations:** ^1^Faculty of Economics and Business, University of Groningen, Groningen, The Netherlands.; ^2^Lifelines Cohort Study and Biobank, Roden, The Netherlands.; ^3^University Medical Center Groningen, Groningen, The Netherlands.; ^4^Aletta Jacobs School of Public Health, University of Groningen, Groningen, The Netherlands.; ^5^Faculty of Law, University of Groningen, Groningen, The Netherlands.

## Introduction

 Tremendous progress has been made in improving human health over the last 150 years, but more recently there have been serious setbacks. Well before the coronavirus disease 2019 (COVID-19) pandemic, increases in life expectancy were levelling off, non-communicable diseases (NCDs) were on the rise worldwide, and health inequalities between socioeconomic and ethnic groups were escalating.^1^

 Reducing NCDs is stipulated in various international instruments as the responsibility of governments. For instance, the United Nations Sustainable Development Goal (SDG) 3.4 aims to “reduce by one-third premature mortality from NCDs through prevention and treatment and promote mental health and well-being [by 2030].” Many countries go on to use such goals to set aspirational public health benchmarks. However, a recent report has concluded that unfortunately, “[a]lthough premature mortality from NCDs is declining in most countries, for most the pace of change is too slow to achieve SDG target 3.4.”

 The rise in NCDs and the ineffectiveness of aspirational benchmarks in countering premature NCD mortality raises the question; How can public health benchmarks be met? We build on an earlier publication (in Dutch) to argue that such benchmarks could be enforced by embedding them in public health legislation.^2^ In doing so, we draw inspiration from environmental regulation where benchmarks have been common practice for a while. We recognize that it concerns two distinct fields of regulation, with environmental law dealing largely with emissions and health law, among other matters, with restrictions on price, place and promotion of unhealthy goods. Yet we argue that the upstream policy tools (eg, mandated benchmarks) that environmental law has at its disposal can provide inspiration for domestic health regulation.

## Lessons From Environmental Regulation

 The environment has been under substantial pressure over the last few decades with global warming affecting people worldwide. There are SDGs for the environment and a host of international treaties call on governments to protect and improve the environment. Thus, it is not surprising that environmental regulation can provide some leads for public health policy.

 It serves to note that the imposition of SDGs and various treaties for the protection of the environment are a consequence of deeper underlying theoretical considerations. A common approach that can be taken is an externality view of pollution. Indeed, the threat to the environment is to a considerable extent a consequence of the fact that environmental damage is insufficiently reflected in market prices and that, therefore, polluting products and procedures are overused. Overarchingly, an important step to remedy this caveat, following Coase,^3^ is to establish environmental property rights. Environmental benchmarks form an example of this as they effectively put the ownership of the environment in the hands of the government, which then has the task to protect the environment. Top level agreements such as the Kyoto and the Paris accords can be seen as an example of such benchmarks.^4^ While the Paris Agreement is rather recent, the effects of the earlier Kyoto Protocol have been carefully scrutinised and the legally binding agreements regarding reduction emissions have reduced emissions substantially.^5^

###  Legislated Emission Benchmarks

 Mandated environmental quality standards have been imposed through international, regional and domestic legislation. For instance, drawing on European Union (EU) legislation, Member States have set legally mandated quantitative and qualitative targets for maximum concentrations of various substances in the air, groundwater and soil.^6^ If target values are exceeded, the government is legally required to take appropriate action to reduce concentrations to below the target values. However, specifying mandatory limit values does not mandate the specific policies and measures to achieve the targets. Governments can choose the solutions that fit the political preferences of the sitting administration. Nevertheless, the target values may exert pressure on the government to do more than they might have done otherwise.

 A case in point is the Dutch Integrated Approach to Nitrogen (*Programma Aanpak Stikstof*, PAS), which is the Dutch Government’s response to consistent exceedance of nitrogen concentration levels in vulnerable nature conservation areas protected by the EU Habitats Directive.^7,8^ In May 2019, the highest Dutch administrative court ruled that PAS was in breach of EU Law.^9^ As a result, the government was forced to introduce new measures to reduce nitrogen deposition in nature conservation areas.

###  Arising From International Treaties

 While domestic legislation is the common route to domestically enforceable benchmarks, in some cases international treaties may be enforced before domestic courts to hold the government accountable.^10,11^ Again, the Netherlands is a case in point. After a long period of litigation by the Urgenda consortium, the Dutch supreme court ruled in 2019 that the Dutch state was not making sufficient effort to curb carbon emissions. This breached the human rights of Dutch residents and violated a host of international environmental treaties ratified by the Dutch government.^12^ As a consequence of the ruling, which coincided with the nitrogen ruling, the Dutch government took extensive steps to curb emissions.^13^

## Right to Health Framework

 Legally mandated public health benchmarks are grounded in the human right to health, as set out in a range of international human rights treaties to which most countries worldwide are bound. The most frequently cited right to health provision is Article 12 of the International Covenant on Economic, Social and Cultural Rights, and its authoritative General Comment 14, setting out the meaning and scope of the right to health.^14^

 General Comment 14 identifies a set of core obligations, that is ‘minimum essential levels of each of the rights.’^15^ One of these core obligations is the obligation on government to ‘adopt and implement a national public health strategy and plan of action, on the basis of epidemiological evidence, addressing the health concerns of the whole population.’ This strategy must include ‘methods, such as right to health indicators and benchmarks, by which progress can be closely monitored.’^16^ Several authors have suggested that these core obligations are non-derogable, that is, they cannot be restricted or limited in any way.^17^ Thus, there are compelling reasons to assume that governments already have the legal obligation to set a public health strategy that includes indicators and benchmarks. Similarly, such domestic benchmarks can also be perceived as national minimum obligations or thresholds.^18,19^

 Before the adoption of General Comment 14, human rights scholars debated the nature and scope of such indicators and benchmarks.^20^ Fukuda-Parr suggested using development-based indicators, disaggregating to various development levels.^21^ Former UN Special Rapporteur on the Right to Health, Paul Hunt, proposed a four-step process that includes states setting their own benchmarks.^22^ More recently, Backman et al proposed a broad range of indicators for health systems, which are mostly process-oriented.^23^ Along the lines of Hunt, we propose setting benchmarks for NCDs specifically by domestic governments, focusing on outcome indicators for NCDs.

## Towards Legally Mandated Public Health Benchmarks

 We suggest that the process of implementing legally mandated public health benchmarks consists of three steps.

###  1. Incorporating Legal Obligations Into Public Health Law

 The first step towards mandated public health benchmarks is to incorporate a legal provision requiring the government to periodically set benchmarks for this purpose.^24^ The exact wording and means to regulate this will depend on the domestic context and approach. Nevertheless, this supposes that the government reports on these targets to parliament and takes appropriate actions to ensure benchmarks are met. Such benchmarks do not necessarily only have to be set at the domestic level, they can also be imposed at sub-national levels, such as states and municipalities.

###  2. Choosing Indicators

 While the Backman et alframework proposes 72 indicators, only a limited set of indicators deals with the health situation in a specific country. Moreover, these indicators deal mainly with mortality instead of morbidity^[1]^. However, morbidity and especially health-related quality of life is a key focus of public health policy in many countries. Therefore, mortality indicators of Backman et alwill need to be supplemented with indicators for morbidity, health-related quality of life and costs. For instance, self-assessed (mental) health, proportion of the population with certain chronic diseases, lifestyle indicators and healthcare expenditures. The indicators can be measured at regional and group level to account for (*a*) the decentralised nature of public health policy; and (*b*) regional, socioeconomic and ethnic health disparities^[2]^.

###  3. Setting Benchmarks

 Complementing indicators, benchmarks need to be set against which these indicators are compared. Such benchmarks can be derived from international standards. In the case of public health, the guiding principle could be SDG 3.4 with its goal to “reduce by one-third premature mortality from NCDs through prevention and treatment and promote mental health and well-being [by 2030].” National governments should set targets for their public health benchmarks to achieve this overarching goal. This goal can be supplemented with more ambitious domestic targets and/or supplemented with targets for reducing health disparities or for the health of specific groups in the population.

 Naturally, implementing the three steps requires a more detailed policy process that goes beyond the scope of the current contribution. Importantly, it requires a broad societal coalition that prepares national and regional policy for the setting, monitoring and acting upon legally mandated public health benchmarks.

## Achieving Public Health Benchmarks Through Litigation

 As well as through regulations, public health benchmarks can be enforced through litigation. As evidenced above, governments worldwide are bound by international health instruments that implicitly or explicitly call for either public health benchmarks in general or for specific benchmarks. These international and domestic obligations to set benchmarks create a basis for litigation. In line with the Urgenda and Shell judgments in the Netherlands,^10-12^ similar procedures could be used to hold national governments accountable for achieving domestic and international benchmarks including the SDGs, and international and regional human rights standards. States committed to the SDGs and to human rights law could be held accountable by domestic courts for failure to introduce policies to achieve these goals.^12^ In this case, even in the absence of specific domestic legislation, mandated public health targets arise.

## Acknowledgments

 We thank Kars de Graaf, Luc Hagenaars, Judith Hessels, Yinjie Zhu, and Scott Burris for their critical and helpful feedback, and Helen West for effective language editing.

## Ethical issues

 Not applicable.

## Competing interests

 Authors declare that they have no competing interests.

## Authors’ contributions

 JOM wrote the first draft of the article, BCAT offered feedback and added content in relation to the legal dimensions. Both authors were intensively involved in (re)writing and reviewing the article. BCAT managed the process of review.

## Endnotes

 [1] Infant mortality rate, mortality rate of children younger than 5 years, Maternal mortality ratio, Life expectancy. [2] See, for instance, the European Core Health Indicators, which are updated with a significant lag and are only available on the national level. National surveillance data tend to also be lagged, and while available on the subnational level often lack international comparability and/or are not available at the public health relevant unit of observation (ie, neighbourhoods and individuals).
